# New Books

**Published:** 2007-10

A Computer Scientist’s Guide to Cell Biology

William W. Cohen

New York:Springer, 2007. 100 pp. ISBN: 978-0-387-48275-0, $29.95

Accounting for Climate Change: Uncertainty in Greenhouse Gas Inventories—Verification, Compliance, and Trading

D. Lieberman, M. Jonas, Z. Nahorski, S. Nilsson, eds.

New York:Springer, 2007. 162 pp. ISBN: 978-1-4020-5929-2, $139.00

Algorithmic Aspects of Bioinformatics

Hans-Joachim Böckenhauer, Dirk Bongartz

New York:Springer, 2007. 396 pp. ISBN: 978-3-540-71912-0, $89.95

Bioinformatics and the Cell: Modern Computational Approaches in Genomics, Proteomics and Transcriptomics

Xuhua Xia

New York:Springer, 2007. 349 pp. ISBN: 978-0-387-71336-6, $129.00

Expert Witnessing and Scientific Testimony: Surviving in the Courtroom

Kenneth S. Cohen

Boca Raton, FL:CRC Press, 2007. 272 pp. ISBN: 978-1-420-05503-0, $99.95

Fate of Pharmaceuticals in the Environment and in Water Treatment Systems

Diana S. Aga

Boca Raton, FL:CRC Press, 2007. 400 pp. ISBN: 1-420-05232-2, $139.95

Fundamentals of Data Mining in Genomics and Proteomics

Werner Dubitzky, Martin Granzow, Daniel P. Berrar, eds.

New York:Springer, 2007. 281 pp. ISBN: 978-0-387-47508-0, $79.95

Handbook for the Chemical Analysis of Plastic and Polymer Additives

Michael Bolgar, Jack Hubball, Joe Groeger, Susan Meronek

Boca Raton, FL:CRC Press, 2007. 504 pp. ISBN: 1-420-04487-7, $249.95

International Seminar on Nuclear War and Planetary Emergencies: 36th Session

R. Ragaini, ed.

Hackensack, NJ:World Scientific Publishing Co., 2007. 440 pp. ISBN: 981-270-922-3, $119.00

Introduction to Computational Biology: An Evolutionary Approach

Bernhard Haubold, Thomas Wiehe

New York:Springer, 2007. 328 pp. ISBN: 978-3-7643-6700-8, $49.95

Java for Bioinformatics and Biomedical Applications

Harshawardhan Bal, Johnny Hujol

New York:Springer, 2007. 342 pp. ISBN: 978-0-387-37235-8, $89.95

Living with the Earth: Concepts in Environmental Health Science, 3rd ed.

Gary S. Moore

Boca Raton, FL:CRC Press, 2007. 559 pp. ISBN: 0-8493-7998-9, $64.95

Models in Environmental Regulatory Decision Making

Committee on Models in the Regulatory Decision Process, National Research Council

Washington, DC:National Academies Press, 2007. 286 pp. ISBN: 978-0-309-11000-6, $56.25

Networks: From Biology to Theory

Jianfeng Feng, Jürgen Jost, Minping Qian, eds.

New York:Springer, 2007. 313 pp. ISBN: 978-1-84628-485-4, $99.00

Nuclear Nebraska: The Remarkable Story of the Little County That Couldn’t Be Bought

Susan Cragin

New York:Amacon Books, 2007. 304 pp. ISBN: 978-8144-7430-3, $24.95

Public Health Advocacy and Tobacco Control

Simon Chapman

Oxford, UK:Blackwell, 2007. 336 pp. ISBN: 978-1-405-16163-3, $59.95

Renewable Energy Cannot Sustain a Consumer Society

Ted Trainer

New York:Springer, 2007. 200 pp. ISBN: 978-1-4020-5548-5, $59.95

Risk Assessment of Chemicals: An Introduction

C.J. van Leeuwen, T.G. Vermeire, eds.

New York:Springer, 2007. 688 pp. ISBN: 978-1-4020-6101-1, $199.00

Saudia Arabia: An Environmental Overview

Peter Vincent

Boca Raton, FL:CRC Press, 2007. 350 pp. ISBN: 978-0-415-41387-9, $179.95

Scientific Basis of Tobacco Product Regulation

Report of a WHO Study Group

Albany, NY:WHO Press, 2007. 120 pp. ISBN: 978-924-120945-3, $27.00

The Political Economy of World Energy

Ferdinand E. Banks

Hackensack, NJ:World Scientific Publishing Co., 2007. 464 pp. ISBN: 981-270-036-6, $98.00

The Statistics of Gene Mapping

David Siegmund, Benjamin Yakir

New York: Springer, 2007. 331 pp. ISBN: 978-0-387-49684-9, $79.95

Veterans and Agent Orange: Update 2006

Committee to Review the Health Effects in Vietnam Veterans of Exposure to Herbicides

Washington, DC:National Academies Press, 2007. 900 pp. ISBN: 978-0-309-10708-2, $129

